# 572. Insurance Changes Are Common and Associated with Delayed Cabotegravir Plus Rilpivirine Injections

**DOI:** 10.1093/ofid/ofaf695.181

**Published:** 2026-01-11

**Authors:** Jennifer M Davis, Sara H Bares, Jennifer O’Neill, Elizabeth Lyden

**Affiliations:** University of Nebraska Medical Center, Omaha, NE; University of Nebraska Medical Center, Omaha, NE; University of Nebraska Medical Center, Omaha, NE; University of Nebraska Medical Center, Omaha, NE

## Abstract

**Background:**

Long-acting cabotegravir plus rilpivirine (LA CAB/RPV) offers an alternative to daily oral antiretroviral therapy, but implementation can be disrupted by insurance-related barriers. We examined the frequency of insurance changes and their impact on injection timing.

**Figure:**
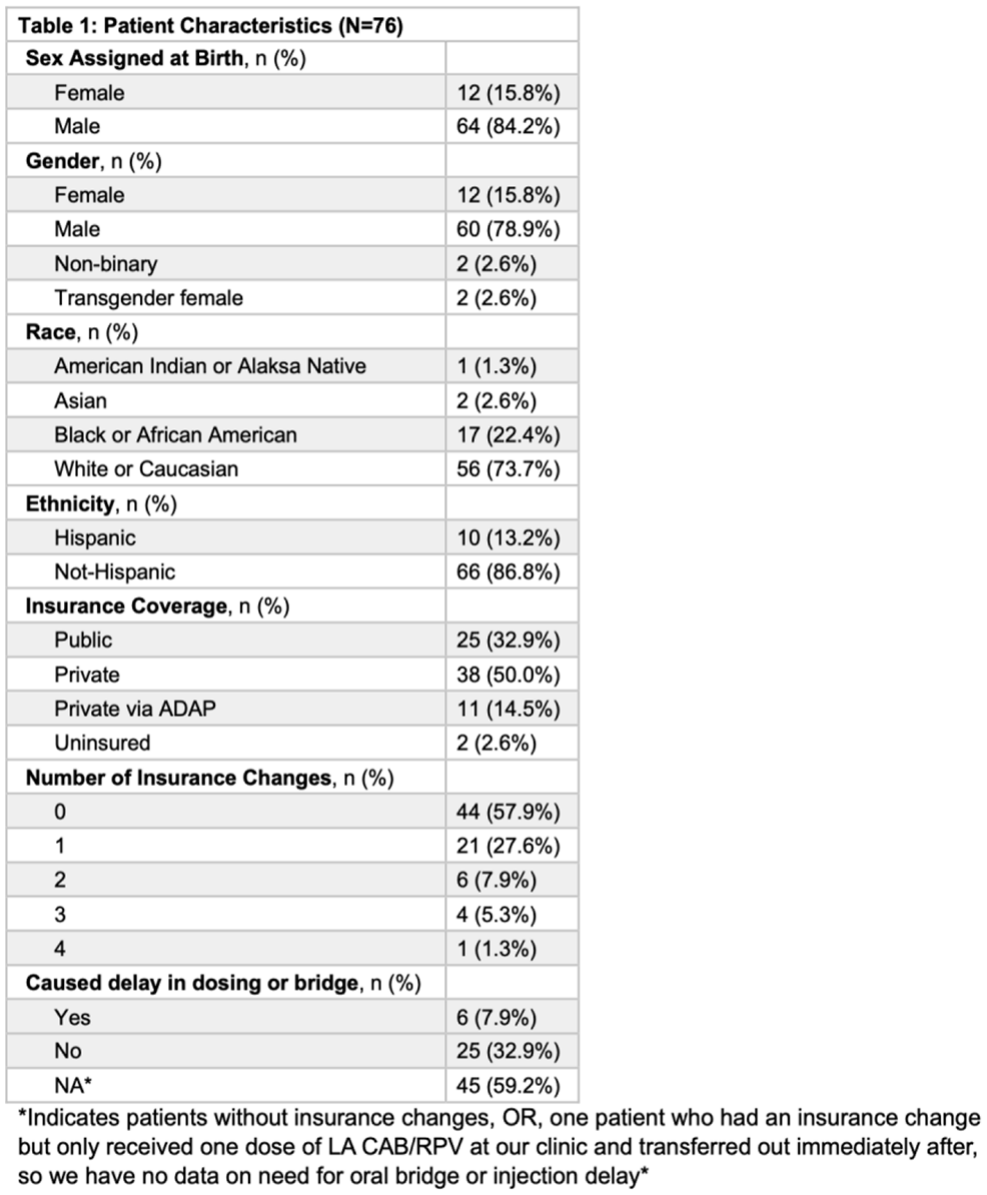

**Figure:**
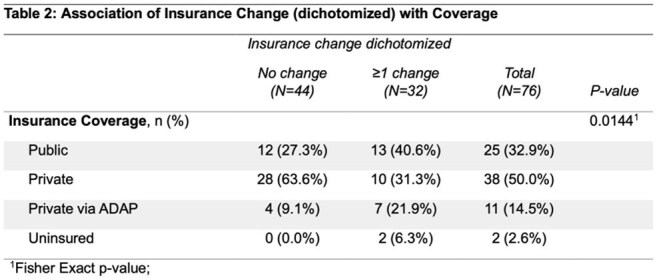

**Methods:**

We conducted a single-center retrospective review of people with HIV receiving LA CAB/RPV at our Midwestern Ryan White clinic between May 4, 2022 (the first commercial injection administered at our clinic) and June 30, 2024. Demographics and clinical characteristics including insurance type and number of insurance changes while on LA CAB/RPV were collected. Fisher’s exact test was used to evaluate associations between insurance type and insurance changes and between insurance change and delayed injections.

**Results:**

A total of 76 patients met the inclusion criteria with median age 42 years, 78.9% cisgender male, 73.7% White, 33% Black, and 13.2% Hispanic/Latinx (Table 1). At time of LA CAB/RPV initiation, 25 (32.9%) had public insurance, 38 (50%) private, 11 (14.5%) AIDS Drug Assistance Program-sponsored private insurance, and 2 (2.6%) were uninsured. Nearly half (32/76 or 42.1%) of patients had an insurance change after LA CAB/RPV initiation. Of the 32 patients with insurance changes, 21 (65.6%) had one, 6 (18.8%) had two, 4 (12.5%) had three, and 1 (3.1%) had four. Those with private insurance at LA CAB/RPV initiation were least likely to have insurance changes compared to other types of insurance (p=0.014) (Table 2). Six patients experienced a delayed injection or switch to an oral bridge while awaiting insurance authorization; no virologic failures occurred. All of the patients who experienced delays or need for an oral bridge had ≥ 1 insurance change compared to only 37.1% of those without a delay (p = 0.0017).

**Conclusion:**

Nearly half of patients receiving LA CAB/RPV experienced insurance changes, which were linked to delayed injections. Clinics implementing LA CAB/RPV must develop systems to proactively address insurance changes in a timely manner to avoid adverse patient outcomes.

**Disclosures:**

Jennifer M. Davis, MD, GSK/ViiV Healthcare: Grant/Research Support Sara H. Bares, MD, Gilead Sciences: Expert Testimony

